# Association Between Plasma Trimethylamine N-Oxide (TMAO) Levels and Subclinical Atherosclerosis in Asymptomatic Adults

**DOI:** 10.7759/cureus.107850

**Published:** 2026-04-27

**Authors:** Tehzeeb Abdul Sattar, Navpreet Batth, Samirah Mohammed Khan, Daud Gul, Eemaz Nathaniel, Nidhi Reji, Jisha Jayanand Sheela, Waqar Ahmed Cheema, Youssef Mohamed Fawzi Moustafa Ahmed, Haya Mohanned M. T. Saffarini

**Affiliations:** 1 Pathology, Liaquat University of Medical and Health Sciences, Jamshoro, PAK; 2 Internal Medicine, Medway Maritime Hospital, Gillingham, GBR; 3 Family Medicine, Dr. Anthony Davies Family Practice, Waterloo, CAN; 4 Medicine, Rehman Medical College, Peshawar, PAK; 5 General Surgery, The Rotherham NHS Foundation Trust, Rotherham General Hospital, Rotherham, GBR; 6 Endocrinology, Good Hope Hospital, University Hospitals Birmingham NHS Foundation Trust, Birmingham, GBR; 7 Medicine, Independent Medical College, Faisalabad, PAK; 8 Medicine, Gulf Medical University, Ajman, ARE

**Keywords:** cardiovascular risk, carotid intima-media thickness, gut microbiota, subclinical atherosclerosis, tmao, trimethylamine n-oxide

## Abstract

Background: Trimethylamine N-oxide (TMAO), a microbiota-derived metabolite in the gut, has recently become a putative biomarker of cardiovascular disease (CVD). Nonetheless, there is little evidence that plasma TMAO levels are linked with increased carotid intima-media thickness (CIMT) in individuals without symptoms. The study aimed to assess the association between plasma TMAO concentration and subclinical atherosclerosis in seemingly healthy adults.

Methods: The study used a hospital-based cross-sectional observational design with asymptomatic adults aged 25 to 60 years without a known history of cardiovascular disease. There was a collection of demographic and clinical data, such as age, sex, body mass index (BMI), smoking status, hypertension, diabetes, and dyslipidemia. Venous blood samples were collected after fasting, and plasma TMAO was measured by enzyme-linked immunosorbent assay (ELISA). CIMT measured by ultrasound was used to assess subclinical atherosclerosis. Correlation and regression analyses were conducted to assess the association between TMAO and CIMT.

Results: There was a strong, positive, and statistically significant correlation between plasma TMAO levels and CIMT (r = 0.771, p < 0.001). TMAO was also associated with age (r = 0.587) and BMI (r = 0.234), with moderate and weak associations, respectively. The multiple regression analysis showed that age had the strongest association with CIMT, followed by TMAO and BMI. Higher TMAO and CIMT levels were observed in males and in individuals with multiple cardiometabolic comorbidities (e.g., hypertension, diabetes, and dyslipidemia), as well as those with higher red meat intake.

Conclusion: Plasma TMAO levels were significantly associated with increased CIMT in asymptomatic adults. These findings suggest a potential link between gut microbiota-derived metabolites and increased CIMT. However, due to the cross-sectional design, causality and predictive utility cannot be established, and longitudinal studies are required.

## Introduction

Globally, cardiovascular diseases (CVD) cause morbidity and mortality, and atherosclerosis is the main cause of both, often remaining asymptomatic over the years till the clinical manifestations occur [1,2]. Subclinical atherosclerosis should therefore be identified early to enable prevention and risk stratification among asymptomatic individuals [3]. Carotid intima-media thickness (CIMT) and arterial plaque are non-invasive measures that provide useful information about early vascular changes and cardiovascular risk [4].

The use of microbiota-derived metabolites in cardiovascular health has also received recent attention, and one of the most captivating molecules is trimethylamine N-oxide (TMAO) [5,6]. TMAO is produced in the liver through the breakdown of the microbiota of the intestines on the nutrients of the diet, like choline and L-carnitine, to create the trimethylamine [7]. Clinical and experimental studies have shown that an increase in TMAO concentration may favor atherogenesis by enhancing foam cell formation, platelet hyperreactivity, and changes in cholesterol metabolism, and that the activity of the gut microbiota is linked to the occurrence of vascular disease [8,9].

Although high levels of TMAO are linked to major cardiovascular events, the relationship between TMAO and early, subclinical atherosclerotic changes in healthy, symptom-free adults remains under-studied [10,11]. Awareness of such a correlation can improve early risk prediction and offer a new means of prevention. The objective of the research was to evaluate the relationship between plasma TMAO levels and subclinical atherosclerosis and thus to clarify the possible involvement of the intestinal microbiota-produced metabolites in the development of cardiovascular disease.

Research gap and novelty

Although elevated plasma TMAO levels have been associated with adverse cardiovascular outcomes, most of the available evidence is derived from studies involving patients with established or suspected cardiovascular disease [6,10]. However, evidence regarding its role in increased CIMT among asymptomatic individuals remains limited and inconsistent [11]. Therefore, it remains unclear whether TMAO can serve as a potential biomarker for subclinical atherosclerosis in otherwise healthy populations.

The existing research is novel because it will establish the relationship between plasma TMAO concentrations and the initial atherosclerotic shift in adults without clinical disease. This research is not only aimed at filling an existing knowledge gap but also at contributing to the interpretation of TMAO as a potential predictive biomarker of early cardiovascular risk in the population, rather than in disease.

Study objective

The primary objective of this study was to investigate the association between plasma TMAO levels and CIMT, a non-invasive marker of subclinical atherosclerosis, in asymptomatic adults aged 25-60 years. Secondary objectives were to evaluate the relationship between TMAO levels and traditional cardiovascular risk factors, including age, BMI, smoking status, hypertension, diabetes mellitus, dyslipidemia, physical activity, and dietary habits.

## Materials and methods

Study framework and setting

It was a hospital-based cross-sectional observational study examining the relationship between subclinical atherosclerosis and plasma TMAO levels in asymptomatic adults. The study was conducted from March 2025 to September 2025 in the outpatient departments of general medicine and cardiology at a single tertiary care hospital. Participants primarily presented for routine health check-ups or minor non-cardiovascular complaints. Demographic information was collected, along with laboratory measurements of plasma TMAO levels and radiological assessment of CIMT as a measure of subclinical atherosclerosis. Individuals with known cardiovascular disease were excluded based on medical history and available clinical records. No additional diagnostic testing for coronary artery disease was performed as part of the study protocol.

Sampling technique

Eligible participants were enrolled through convenience sampling from outpatient clinics presenting for routine health check-ups or minor non-cardiovascular complaints. Participants who satisfied the eligibility criteria were enrolled sequentially until the target sample size was achieved. The minimum required sample size was estimated using an anticipated moderate correlation (r = 0.25-0.30), informed by prior studies demonstrating associations between TMAO and vascular markers such as CIMT [12,13], with a significance level of 0.05 and statistical power of 80%, yielding an approximate sample size of 280-300 participants. To improve statistical precision and account for potential missing data, 402 participants were included in the final analysis.

Study population

**Figure 1:**
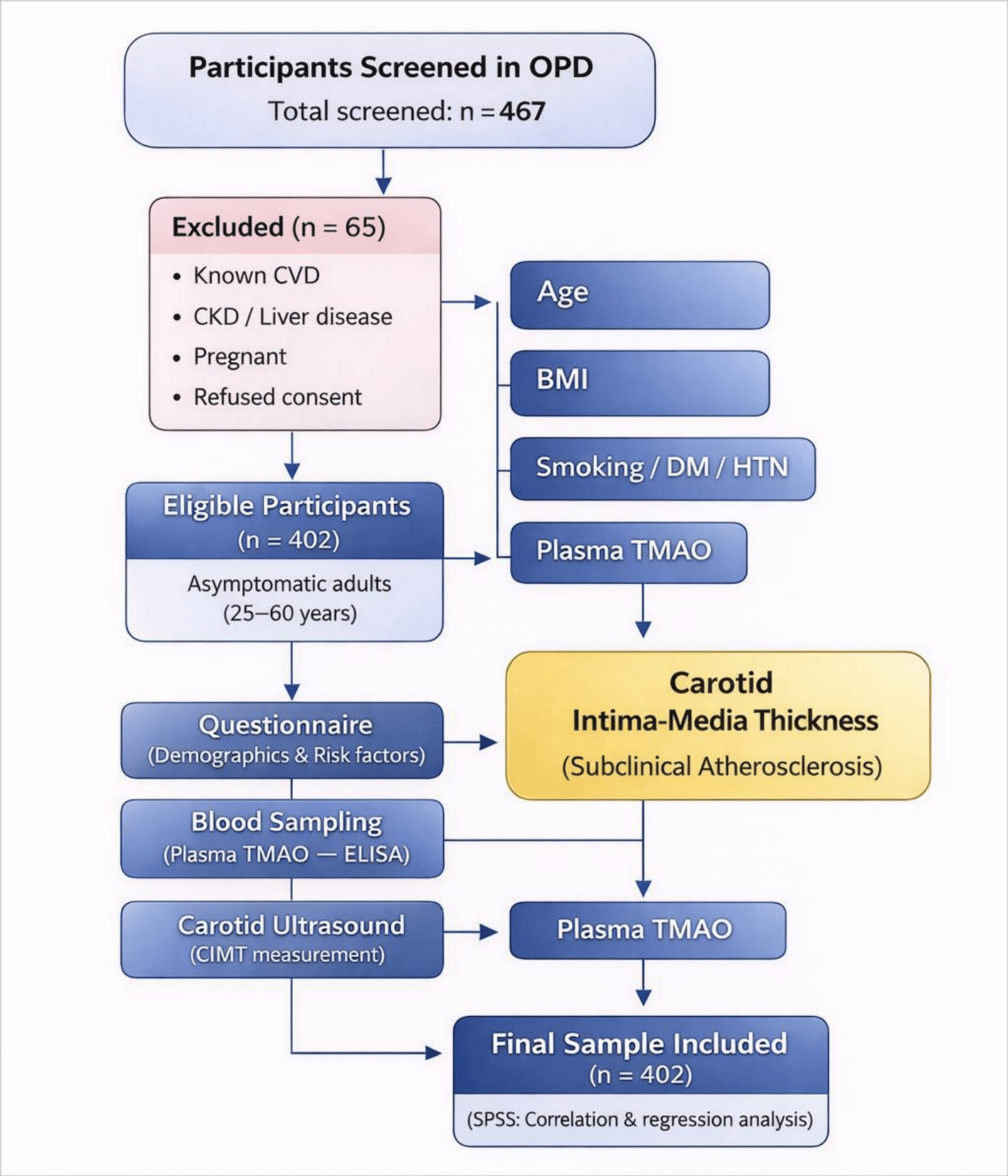
Study flow diagram of participant selection, data collection, and analysis A total of 467 participants were screened, with 65 excluded based on predefined criteria. The final sample included 402 asymptomatic adults. Data collection involved questionnaires, plasma TMAO measurement (ELISA), and CIMT assessment by ultrasound. Associations were analyzed using correlation and regression models. TMAO: Trimethylamine N-oxide, CIMT: carotid intima-media thickness, ELISA: enzyme-linked immunosorbent assay, OPD: outpatient department, CVD: cardiovascular disease, CKD: chronic kidney disease, DM: diabetes mellitus, HTN: hypertension, SPSS: SPSS Statistics (IBM Corp., Armonk, NY, USA)

The study participants were asymptomatic adults aged 25-60 years with no prior diagnosis of cardiovascular disease. Participants were recruited from the general medicine and cardiology outpatient departments, where they primarily presented for routine health check-ups or minor non-cardiovascular complaints. “Asymptomatic” was defined as the absence of clinically evident cardiovascular symptoms such as chest pain or dyspnea on exertion, and no prior diagnosis of coronary artery disease, stroke, or peripheral vascular disease, based on self-report and available medical records. Individuals with known cardiovascular disease were excluded. Chronic kidney disease was defined as an estimated glomerular filtration rate (eGFR) <60 mL/min/1.73 m² [14], and individuals with such an eGFR were excluded. Additional exclusion criteria included chronic liver disease, pregnancy, and any condition known to significantly affect TMAO metabolism or vascular structure. Information regarding hypertension, diabetes mellitus, and dyslipidemia was obtained through self-report and verified, where possible, using medical records. Hypertension and diabetes mellitus were defined based on prior clinical diagnosis or current use of relevant medications, verified through self-report and available medical records. Dyslipidemia was defined as a prior clinical diagnosis, use of lipid-lowering medications, or documented abnormal lipid profile (elevated low-density lipoprotein (LDL) cholesterol, triglycerides, or reduced high-density lipoprotein (HDL) cholesterol). Participants were clinically assessed by physicians in the outpatient departments, and eligibility was determined based on clinical evaluation, history, and available medical records. No additional laboratory or imaging investigations (e.g., liver function tests, kidney function tests, or abdominal ultrasound) were performed as part of the study protocol; available clinical records were used where applicable. The overall study workflow, including participant selection, data collection, and analysis, is illustrated in Figure 1.

Data collection

Demographic and Clinical Characteristics

The structured questionnaire was used to acquire demographic and clinical information. The age, gender, height, and weight were recorded and the body mass index (BMI) was calculated. Modifiable lifestyle variables, including smoking status and the amount of physical activity, were included because of their impact on cardiovascular risk. Physical activity levels were assessed using a self-reported questionnaire and categorized into four groups based on the frequency and intensity of activity: sedentary (no regular physical activity), light activity (occasional low-intensity activity), moderate activity (regular activity such as brisk walking for at least 150 minutes per week), and vigorous activity (high-intensity exercise such as running or sports for at least 75 minutes per week). This classification was adapted from standard physical activity guidelines. The participants also inquired about the history of the known cardiovascular risk factors such as hypertension, diabetes mellitus, and dyslipidemia. The demographic questionnaire was used to collect dietary data. Dietary intake was assessed using a structured, self-reported frequency questionnaire focusing on the consumption of TMAO-related food groups, including red meat, eggs, and fish. Participants were asked to report the frequency of consumption (e.g., rarely/never, one to two times per week, three to four times per week, five or more times per week). The highest reported frequency was used to categorize participants for analysis of the influence of diet on plasma TMAO levels. The questionnaire was administered in a standardized manner by trained personnel to ensure consistency in data collection. Wherever possible, responses were cross-verified with available medical records to improve accuracy.

Blood sample collection

All respondents were asked to fast overnight for eight to 12 hours prior to blood collection, and a 5 mL venous blood sample was collected using standard aseptic practices. The blood samples were placed in the respective collection tubes and sent to the laboratory for processing. The samples were centrifuged, and the plasma was kept at −80°C until further biochemical tests were conducted. All samples were processed within a standardized time frame to minimize pre-analytical variability. Storage and handling protocols were strictly followed to preserve sample integrity.

Measurement of plasma TMAO levels

An enzyme-linked immunosorbent assay (ELISA) kit (Elabscience, Wuhan, China) was used to determine plasma TMAO concentration according to the manufacturer's instructions. The plasma samples were added to ELISA plates and incubated with reagents to allow antigen-antibody interactions. They incubated and washed it, then performed a colorimetric reaction and measured the optical density on a microplate reader. TMAO levels were determined using a standard calibration curve and expressed in micromoles per liter (µmol/L). All samples were measured under standard laboratory conditions to assess measurement reliability. Although liquid chromatography-mass spectrometry (LC-MS/MS) is considered the gold standard for TMAO quantification, ELISA was selected given the study setting's feasibility and resource constraints. All samples were analyzed in duplicate, and average values were used for final analysis to enhance measurement reliability.

Assessment of subclinical atherosclerosis

CIMT, measured by high-resolution B-mode ultrasonography using a Toshiba Xario ultrasound system (Canon Medical Systems, Ōtawara, Japan) equipped with a linear-array transducer (7-12 MHz), was used to assess subclinical atherosclerosis. CIMT measurements were obtained from the far wall of the common carotid artery approximately 1 cm proximal to the bifurcation. The participants were examined in the supine position, and the measurements were taken at the left and right carotid arteries. Multiple measurements were used to conduct the analysis to increase precision. Carotid plaques were not measured when present, so as to provide a standard CIMT. All ultrasound examinations were performed in accordance with usual practices by trained staff. To ensure measurement consistency, CIMT assessments were performed by trained sonographers using standardized protocols. Multiple readings from both carotid arteries were averaged to reduce measurement variability. However, intra- and inter-observer variability were not formally assessed.

Outcomes assessment

The primary research outcome was the association between plasma TMAO levels and CIMT, an early marker of subclinical atherosclerosis. Secondary analyses were conducted to assess the relationship between TMAO and cardiovascular risk factors, including age, BMI, smoking status, physical activity, hypertension, diabetes mellitus, and dietary habits.

Statistical analysis

The data was analyzed with SPSS Statistics version 26 (IBM Corp., Armonk, NY, USA). Continuous variables were described using the mean and standard deviation (SD), and categorical variables were described using frequencies and percentages. The Shapiro-Wilk test was used to test the normality of continuous variables. Nonparametric tests were applied where necessary because most variables were not normally distributed. The associations between plasma TMAO levels and CIMT and other continuous variables (i.e., age and BMI) were measured using Spearman's rank correlation analysis. To determine independent predictors of CIMT, multiple linear regression was performed with CIMT as the dependent variable and plasma TMAO, age, BMI, smoking status, and diabetes mellitus as independent variables. Variance inflation factor (VIF) was determined to assess multicollinearity among the associated factor variables. A VIF value <5 was considered indicative of no significant multicollinearity. For group comparisons, the Mann-Whitney U test was used to assess differences in plasma TMAO levels and CIMT between two groups (e.g., gender, hypertension, diabetes, and dyslipidemia). The Kruskal-Wallis H test was used to compare differences across more than two groups (e.g., smoking status, age groups, and dietary categories). Any p-value below 0.05 was regarded as a statistically significant value.

Ethical considerations

The study was carried out in compliance with the principles mentioned in the Declaration of Helsinki. The study was conducted under the ethical permission of the Institutional Review Board (IRB) of Shifa International Hospital (IRB#0301-25-A) of the concerned institution. All the participants had informed consent before enrolment. The participants were assigned a study identification code that guaranteed their confidentiality, and all data were deposited in password-protected files that were only available to the research team.

## Results

Table 1 presents the participants' demographics and clinical characteristics as frequencies (N = 402). The age brackets with the highest participation were 50-59 years (N = 131), 40-49 years (N = 109), and 30-39 years (N = 89); the lowest were those aged 60 years and above (N = 10). There were 209 females and 193 males in the sample, with biological sex comparable between groups. Most of the respondents were married (N = 257), 65 were single, 65 were divorced, and 15 were widowed. In terms of educational attainment, college or university education (N = 186), secondary education (N = 110), and postgraduate education (N = 73) had the highest scores, and 33 had primary education. In terms of occupation, 192 were employed, 94 were self-employed, 60 were homemakers, 33 were unemployed, and 23 were students. Over half (N = 215) did not smoke at all, 94 were smokers, and 93 were ex-smokers. Regarding physical activity, light physical activity (N = 136), sedentary lifestyle (N = 108), moderate activity (N = 106), and 52 respondents were regular participants in vigorous exercise. In terms of clinical conditions, 101 participants had hypertension, 52 had diabetes mellitus, and 103 had dyslipidemia. Eating patterns revealed that the most common frequency was one to two times per week for red meat (N = 152), eggs (N = 155), and fish (N = 190).

**Table 1:** Frequency Distribution of Demographic and Clinical Variables (N = 402) f = frequency; % = valid percentage. N = 402; no missing values for any variable.

Variable	f	%
Age Group Category		
20–29 years	63	15.7
30–39 years	89	22.1
40–49 years	109	27.1
50–59 years	131	32.6
60 years or older	10	2.5
Biological Sex		
Male	193	48.0
Female	209	52.0
Marital Status		
Single	65	16.2
Married	257	63.9
Divorced	65	16.2
Widowed	15	3.7
Educational Attainment		
Primary Education	33	8.2
Secondary Education	110	27.4
College / University	186	46.3
Postgraduate	73	18.2
Occupation		
Student	23	5.7
Employed	192	47.8
Self-employed	94	23.4
Unemployed	33	8.2
Homemaker	60	14.9
Smoking Status		
Never Smoker	215	53.5
Current Smoker	94	23.4
Former Smoker	93	23.1
Physical Activity Level		
Sedentary	108	26.9
Light Physical Activity	136	33.8
Moderate Physical Activity	106	26.4
Regular Vigorous Exercise	52	12.9
Hypertension		
No	301	74.9
Yes	101	25.1
Diabetes Mellitus		
No	350	87.1
Yes	52	12.9
Dyslipidemia		
No	299	74.4
Yes	103	25.6
Red Meat Consumption		
Rarely / Never	87	21.6
1–2 times per week	152	37.8
3–4 times per week	113	28.1
≥5 times per week	50	12.4
Egg Consumption		
Rarely / Never	70	17.4
1–2 times per week	155	38.6
3–4 times per week	131	32.6
≥5 times per week	46	11.4
Fish Consumption		
Rarely / Never	73	18.2
1–2 times per week	190	47.3
3–4 times per week	119	29.6
≥5 times per week	20	5.0

Table 2 indicates that the study sample comprised 402 participants, with a mean age of 43.30 ± 10.85 years (25-60 years). The mean height and weight were 167.74 cm ±8.69 and 70.18 kg ±13.51, respectively, and the mean BMI was 24.83 ±3.68 kg/m 2, which is within the normal-to-overweight range. Plasma TMAO ranged from 2.54 to 12.02 µmol/L, with a mean of 7.32 and a SD of 1.68, indicating moderate variation in this gut microbiota-derived metabolite. CIMT, a measure of subclinical atherosclerosis, ranged from 0.51 to 1.10 mm, with a mean of 0.85 ±0.16 mm, indicating mild atherosclerosis of the carotid artery walls in the population. All in all, these descriptive statistics can provide a baseline profile of the participants' demographic, anthropometric, and vascular features.

**Table 2:** Descriptive Statistics for Continuous Variables (N = 402) M = mean; SD = standard deviation; BMI = body mass index; TMAO = trimethylamine N-oxide; CIMT = carotid intima-media thickness

Variable	N	Min	Max	M	SD	Variance
Age (years)	402	25	60	43.30	10.85	117.76
Height (cm)	402	150.0	190.0	167.74	8.69	75.43
Weight (kg)	402	50.0	110.0	70.18	13.51	182.51
BMI (kg/m²)	402	18.0	36.0	24.83	3.68	13.57
Plasma TMAO (µmol/L)	402	2.54	12.02	7.32	1.68	2.81
CIMT (mm)	402	0.51	1.10	0.85	0.16	0.03

Table 3 shows the results of the normality tests for continuous variables. The Shapiro-Wilk test indicated that most variables, including age, height, weight, CIMT, and BMI, are not normally distributed (p < 0.05). Conversely, the plasma TMAO levels fulfilled the normality assumption (p = 0.679). According to these findings, TMAO was normally distributed, but the other variables were non-normally distributed.

**Table 3:** Tests of Normality for Continuous Variables using Shapiro-Wilk Lilliefors significance correction applied. Normality assessed using the Shapiro-Wilk test (p > .05 = normal distribution). Plasma TMAO was the only variable satisfying normality. BMI = body mass index; TMAO = trimethylamine N-oxide; CIMT = carotid intima-media thickness

Variable	Statistic	df	p	Normal?
Age (years)	0.938	402	0.000	No
Height (cm)	0.986	402	0.001	No
Weight (kg)	0.964	402	0.000	No
BMI (kg/m²)	0.984	402	0.000	No
Plasma TMAO (µmol/L)	0.997	402	0.679	Yes
CIMT (mm)	0.962	402	0.000	No

**Figure 2:**
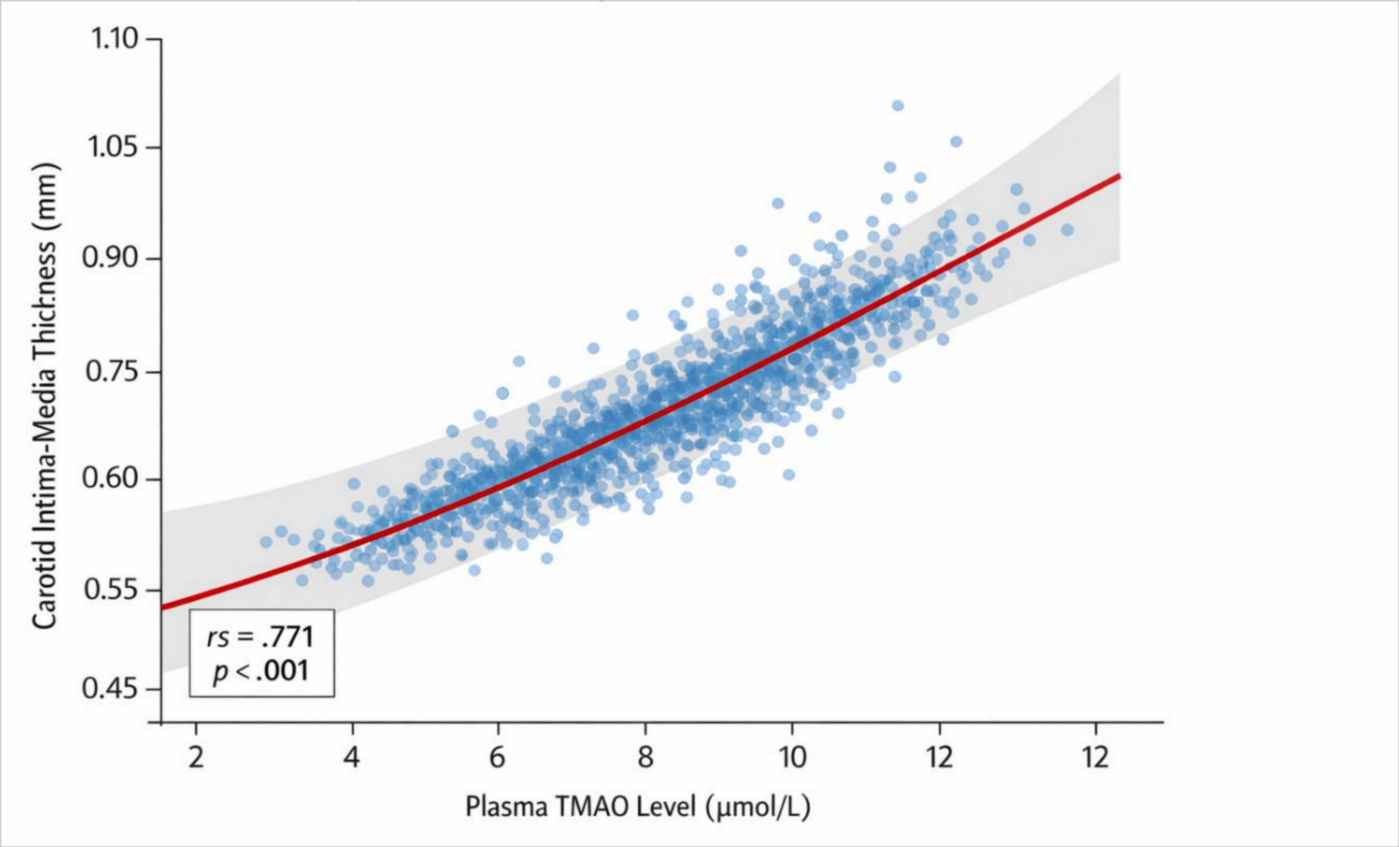
Association Between Plasma TMAO Levels and CIMT Scatter plot illustrating the association between plasma TMAO levels and CIMT in asymptomatic adults. Each point represents an individual participant (N = 402), with a positive linear trend observed (ρ = 0.771, p < 0.001). TMAO = trimethylamine N-oxide; CIMT = carotid intima-media thickness

Figure 2 shows that plasma TMAO levels are correlated with CIMT in the study participants (N = 402). The scatter plot shows a high positive association between plasma TMAO and CIMT (r = 0.771, p = .001), indicating that higher TMAO concentrations are associated with larger CIMT. The increasing slope of the regression line also helps affirm this correlation, indicating that higher plasma TMAO concentrations are more likely to be associated with higher CIMT. This trend suggests that elevated TMAO levels may be associated with greater subclinical atherosclerotic alterations in the carotid arteries.

Table 4 shows Spearman correlation coefficients between plasma TMAO, CIMT, age, and BMI. Plasma TMAO showed a strong positive correlation with CIMT (r = 0.771, p < 0.001). TMAO also demonstrated a moderate positive correlation with age (r = 0.587, p < 0.01) and a weak positive correlation with BMI (r = 0.234, p < 0.01). There was also a strong positive correlation between CIMT and age (r = 0.879, p < 0.01) and a weak positive correlation with BMI (r = 0.140, p < 0.01). In contrast, BMI did not demonstrate a significant correlation with age (r = −0.013). These findings indicate that higher plasma TMAO levels and increasing age are associated with greater CIMT values.

**Table 4:** Spearman's Rho Correlation Matrix for Plasma TMAO, CIMT, Age, and BMI Spearman's correlation coefficients (r) were used because most variables were non-normally distributed. ** p < .01 (two-tailed). BMI = body mass index; TMAO = trimethylamine N-oxide; CIMT = carotid intima-media thickness

Variable	Plasma Trimethylamine N-Oxide (µmol/L)	Carotid Intima-Media Thickness (mm)	Age (years)	BMI
1. Plasma TMAO (µmol/L)	-	.771**	.587**	.234**
2. CIMT (mm)	.771**	-	.879**	.140**
3. Age (years)	.587**	.879**	-	-.013
4. BMI (kg/m²)	.234**	.140**	-.013	-

Table 5 presents the results of the multiple linear regression analysis of predictors of CIMT. The general model was statistically significant (R² = .892, p < .001), indicating that the model explained a high proportion of variance in CIMT, largely driven by the strong association between age and CIMT. Age was the most significant predictor (β = 0.684, p <.001), followed by plasma TMAO levels (β = 0.322, p <.001). The levels of BMI (β= 0.087, p <.001) and diabetes (β= 0.083, p <.001) were also significantly positively related to CIMT, whereas smoking status (β= 0.042, p =.013) was an even smaller, though statistically significant, factor. The VIF values showed no evidence of multicollinearity between the associated factor. These results indicate that higher age, plasma TMAO, BMI, diabetes, and smoking were significantly associated with increased CIMT after adjustment. The high R² should be interpreted with caution, as age demonstrated a particularly strong correlation with CIMT in this dataset

**Table 5:** Multiple Linear Regression Identifying Predictors of Carotid Intima-Media Thickness (CIMT) Dependent variable: CIMT (mm). R = .945; R² = .892; Adjusted R² = .891; F(5, 396) = 655.75, p < .001. B = unstandardized coefficient; SE B = standard error; β = standardized coefficient; VIF = variance inflation factor, BMI = body mass index; TMAO = trimethylamine N-oxide

Predictor	B	SE B	β	t	p	VIF
(Constant)	0.072	0.022	—	3.314	.001	—
Plasma TMAO (µmol/L)	0.031	0.002	0.322	14.257	0.000	1.878
Age (years)	0.010	0.000	0.684	32.102	0.000	1.669
BMI (kg/m²)	0.004	0.001	0.087	4.975	0.000	1.121
Smoking Status	0.008	0.003	0.042	2.484	.013	1.065
Diabetes (Yes)	0.040	0.008	0.083	4.847	0.000	1.087

Table 6 shows the Mann-Whitney U test results for plasma TMAO and CIMT across various groups. The findings revealed that TMAO and CIMT were significantly higher in males than in females (p < .05). The hypertensive participants had much higher mean ranks for TMAO and CIMT than the normative participants (p < .001). Equally, diabetic participants had very high levels of TMAO and CIMT as compared to those who are non-diabetic (p < .001). The dyslipidemic participants also showed much higher levels than non-dyslipidemic participants (p < .001). In general, the results suggest that TMAO and CIMT levels are higher in patients with cardiometabolic risk factors.

**Table 6:** Mann-Whitney U Test: Group Comparisons of Plasma TMAO and CIMT Mann-Whitney U test used due to non-normal distribution. p-values are two-tailed. All results are significant. BMI = body mass index; TMAO = trimethylamine N-oxide; CIMT = carotid intima-media thickness

Variable	Group	n	Mean Rank	U Value (Mann–Whitney U Test)	Z value (Mann–Whitney U test)	p
TMAO by Gender	Male	193	228.10	15,035.5	−4.410	0.000
	Female	209	176.94			
CIMT by Gender	Male	193	218.88	16,814.0	−2.882	.004
	Female	209	185.45			
TMAO by Hypertension	No	301	180.26	8,807.5	−6.327	0.000
	Yes	101	264.80			
CIMT by Hypertension	No	301	174.94	7,205.5	−7.913	0.000
	Yes	101	280.66			
TMAO by Diabetes	No	350	191.68	5,664.0	−4.395	0.000
	Yes	52	267.58			
CIMT by Diabetes	No	350	189.78	4,998.0	−5.247	0.000
	Yes	52	280.38			
TMAO by Dyslipidemia	No	299	189.46	11,800.0	−3.538	0.000
	Yes	103	236.44			
CIMT by Dyslipidemia	No	299	186.78	10,996.0	−4.329	0.000
	Yes	103	244.24			

Table 7 shows the results of the Kruskal-Wallis test of plasma TMAO and CIMT between various groups. Plasma TMAO levels differed significantly by smoking status (p < .001), with the highest mean ranks observed in current smokers, former smokers, and never smokers. CIMT, however, did not significantly differ by the smoking status (p =.420). There was a significant difference between age groups in both TMAO and CIMT (p < .001), with mean ranks increasing with age. Moreover, TMAO levels differed significantly by red meat consumption (p < .001), with higher mean ranks among participants who more often ate red meat. These results indicate that age, smoking, and dietary patterns are associated with differences in plasma TMAO levels; age is the strongest predictor of CIMT.

**Table 7:** Kruskal-Wallis Test: TMAO and CIMT Across Multiple Groups The Kruskal-Wallis H test is used to compare 3 or more independent groups. p-values are two-tailed. CIMT by Smoking Status was not statistically significant (p = .420). TMAO = trimethylamine N-oxide; CIMT = carotid intima-media thickness

Variable	Group	n	Mean Rank	Kruskal–Wallis H Test	df	p
TMAO by Smoking Status	Never Smoker	215	181.30	18.568	2	0.000
	Current Smoker	94	242.85			
	Former Smoker	93	206.40			
CIMT by Smoking Status	Never Smoker	215	194.45	1.736	2	.420
	Current Smoker	94	211.14			
	Former Smoker	93	208.06			
TMAO by Age Group	20–29 years	63	87.92	145.993	4	0.000
	30–39 years	89	144.35			
	40–49 years	109	216.37			
	50–59 years	131	271.97			
	60+ years	10	340.40			
CIMT by Age Group	20–29 years	63	46.90	306.866	4	0.000
	30–39 years	89	111.73			
	40–49 years	109	215.55			
	50–59 years	131	311.90			
	60+ years	10	375.05			
TMAO by Red Meat Consumption	Rarely / Never	87	156.30	46.326	3	0.000
	1–2 times/week	152	179.63			
	3–4 times/week	113	234.13			
	≥5 times/week	50	272.87			

## Discussion

The current study assessed the correlation between plasma TMAO and subclinical atherosclerosis in asymptomatic adults, using CIMT as an indicator of early vascular alterations. Our analysis revealed that plasma TMAO was positively associated with CIMT, moderately correlated with age, and weakly correlated with BMI, indicating that higher TMAO levels were associated with greater CIMT, older age, and slightly with adiposity. The results are consistent with earlier reports showing high TMAO-CIMT correlations, age-dependent increases in TMAO, and weak associations with BMI or adiposity, collectively suggesting that TMAO is associated with subclinical atherosclerosis and cardiometabolic risk in this cohort [12,13]. CIMT also demonstrated a very strong relation with age, a weak relation with BMI, and no significant relation between age and BMI, as carotid thickness is largely age-related [15-17]. However, the relationship between TMAO and subclinical atherosclerosis may not be uniform across different populations and risk groups, particularly among low-risk or apparently healthy individuals. This indicates that the observed association may be context-dependent, and therefore, the findings of the present study should be interpreted as exploratory.

Multiple linear regression revealed that age had the strongest association for CIMT, with plasma TMAO and BMI also statistically significant, indicating that carotid thickness increased significantly with age and moderately with TMAO and slightly with BMI. These results are consistent with previous literature that has found TMAO as an independent variable, age as the major determinant, and BMI as a significant but less significant association with subclinical atherosclerosis [12,13]. Smoking had a small yet significant positive association with CIMT, whereas diabetes had a slightly greater positive association. The results are consistent with other studies showing that current and former smokers exhibit greater CIMT and that patients with type 2 diabetes have higher carotid thickness than healthy controls, indicating that these are independent predictors of subclinical atherosclerosis [18,19]. The regression model accounted for a significant proportion of the variability in CIMT (R² = 0.892), but the correlation between age and CIMT can largely explain this. Age is an established predictor of arterial wall thickening and vascular remodelling, and prior population-based studies have shown strong correlations between age and CIMT. Thus, the model's significant explanatory power is likely due to the prominent role of age in vascular structural modifications, as well as the effects of TMAO and other cardiometabolic risk factors. The relatively high R² observed in the regression model may largely reflect the strong relationship between age and CIMT. Age is a well-established determinant of vascular structural changes, and its dominant contribution in the model may have inflated the overall explained variance. Therefore, the independent contribution of other variables, including TMAO, should be interpreted with caution.

Sex differences were also observed, with males showing much higher plasma TMAO (p = 0.001) and CIMT (p = 0.004) than females, consistent with the literature indicating that men develop subclinical atherosclerosis earlier than women [20,21]. Hypertensive subjects also exhibited significantly elevated TMAO and CIMT compared to normotensive subjects (p = 0.001), consistent with other studies showing that increased TMAO is associated with endothelial dysfunction and thicker carotid arteries in hypertension [22,23]. In the same manner, diabetic individuals exhibited markedly higher TMAO and CIMT than non-diabetic individuals (p < 0.001), thereby validating the relationship between type 2 diabetes, increased TMAO, and carotid thickening [24,25]. TMAO was found to be higher and the carotid arteries thicker in dyslipidemic individuals than in non-dyslipidemic individuals, consistent with other studies that found TMAO to be associated with metabolic syndrome and lipid disorders. Dyslipidemia is linked to CIMT [25,26].

Current smokers had significantly higher levels of TMAO than former and never smokers. Still, current smokers had no significant differences in CIMT compared to former and never smokers, in agreement with previous research that smoking raises TMAO but with some inconsistent effects on carotid thickness [27,28]. Plasma TMAO and CIMT rose with age, with the maximum observed in older participants, and this is supported by previous literature on age-related TMAO accumulation and linear age-related increments in CIMT [12,29]. Lastly, plasma TMAO increased with higher red meat intake, a diet-related finding, consistent with reports that TMAO levels rise as people consume more red meat, especially when intestinal microbiota composition predicts TMAO levels [30].

In general, the results of the present research indicate that plasma TMAO concentrations are correlated with increased CIMT and multiple cardiometabolic risk factors. Increased levels of TMAO were especially found in older adults, men, and those with hypertension, diabetes, dyslipidemia, and increased intake of red meat. The study findings support the hypothesis that TMAO is an outcome of interactions among metabolic, lifestyle, and dietary factors and may reflect underlying metabolic and vascular alterations associated with cardiovascular risk and subclinical atherosclerosis. However, these findings should be interpreted cautiously due to the cross-sectional design and potential confounding factors. Furthermore, while established conditions such as hypertension, diabetes, and dyslipidemia are well-recognized risk factors for atherosclerotic cardiovascular disease, TMAO should be considered a potential risk marker rather than a confirmed causal risk factor, given the limited evidence that reducing TMAO reduces atherosclerosis.

This study has several strengths. It includes a relatively large sample of asymptomatic adults and uses standardized, non-invasive measures such as CIMT, along with biochemical assessment of plasma TMAO levels. Additionally, multiple cardiometabolic variables were evaluated, allowing a broader assessment of factors associated with subclinical atherosclerosis.

Study limitations

Several limitations to this study should be considered when interpreting the findings. First, the cross-sectional design does not allow for the establishment of causal relationships between plasma TMAO levels and subclinical atherosclerosis. Longitudinal studies are required to determine whether elevated TMAO levels contribute to the Development of atherosclerotic changes. Second, participants were recruited through convenience sampling from a single tertiary care hospital in Islamabad, Pakistan, which may limit external validity and generalizability to the broader population. Third, although CIMT is a widely used non-invasive marker of subclinical atherosclerosis, it does not capture the full spectrum of vascular pathology, including plaque composition or instability.

Fourth, the final regression model did not include certain important confounding variables, such as sex, hypertension, dyslipidemia, and detailed dietary factors, to minimize the risk of overfitting given the number of predictors relative to the sample size. However, the exclusion of these variables may have resulted in residual confounding, potentially influencing the observed associations between TMAO and CIMT; therefore, the regression findings should be interpreted with caution. Also, the high R² observed in the regression model may reflect the dominant influence of age, and potential model overfitting cannot be completely excluded.

In addition, plasma TMAO levels were measured using ELISA rather than LC-MS/MS, which may introduce variability in measurement. Lastly, other microbiota-related metabolites and inflammatory biomarkers were not assessed, which may have provided further insight into the underlying mechanisms of vascular changes.

Future directions

Longitudinal cohort studies are needed to evaluate further the temporal relationship between plasma TMAO levels and the progression of subclinical atherosclerosis. To encourage generalizability, larger population-based studies in other groups would be desirable to corroborate the findings. In addition, more sophisticated vascular imaging modalities (e.g., coronary artery calcium scores or plaque imaging) would provide more informative insights into the relationship between TMAO and cardiovascular pathology. In addition, future research into the mechanisms of the gut microbiota, diet, and metabolic processes in TMAO formation can identify therapeutic targets to reduce cardiovascular risk.

## Conclusions

This study demonstrates a significant association between plasma TMAO levels and CIMT in asymptomatic adults. Higher TMAO levels were observed alongside increased CIMT, suggesting a potential link between gut microbiota-derived metabolites and subclinical atherosclerosis. However, due to the cross-sectional design, causal relationships cannot be established. TMAO may represent a potential marker of subclinical atherosclerosis; however, further longitudinal studies are required to clarify its predictive value and role in cardiovascular risk assessment.
